# Study of the Emergency Braking Test with an Autonomous Bus and the sEMG Neck Response by Means of a Low-Cost System

**DOI:** 10.3390/mi11100931

**Published:** 2020-10-13

**Authors:** Sergio Fuentes del Toro, Silvia Santos-Cuadros, Ester Olmeda, José Luis San Román

**Affiliations:** 1 Mechanical Engineering Department, Universidad Carlos III de Madrid, Avda. de la Universidad 30, 28911 Leganés, Spain; ssantos@ing.uc3m.es (S.S.-C.); eolmeda@ing.uc3m.es (E.O.); jlsanro@ing.uc3m.es (J.L.S.R.); 2 Institute for Automotive Vehicle Safety (ISVA), Universidad Carlos III de Madrid, Avda. de la Universidad 30, 28911 Leganés, Spain

**Keywords:** electromyography, low-cost, biomechanics, neck injury, autonomous braking

## Abstract

Nowadays, due to the advances and the increasing implementation of the autonomous braking systems in vehicles, the non-collision accident is expected to become more common than a crash when a sudden stop happens. The most common injury in this kind of accident is whiplash or cervical injury since the neck has high sensitivity to sharp deceleration. To date, biomechanical research has usually been developed inside laboratories and does not entirely represent real conditions (e.g., restraint systems or surroundings of the experiment). With the aim of knowing the possible neck effects and consequences of an automatic emergency braking inside an autonomous bus, a surface electromyography (sEMG) system built by low-cost elements and developed by us, in tandem with other devices, such as accelerometers or cameras, were used. Moreover, thanks to the collaboration of 18 participants, it was possible to study the non-collision effects in two different scenarios (braking test in which the passenger is seated and looking ahead while talking with somebody in front of him (BT1) and, a second braking test where the passenger used a smartphone (BT2) and nobody is seated in front of him talking to him). The aim was to assess the sEMG neck response in the most common situations when somebody uses some kind of transport in order to conclude which environments are riskier regarding a possible cervical injury.

## 1. Introduction

Neck injuries caused by non-collision accidents remain a meaningful health problem. Furthermore, these musculoskeletal disorders are expected to continue growing due to the increase of the automatic braking systems in vehicles, as well as the autonomous vehicles. The cervical spine is indeed one of the most sensitive body regions to sudden changes in velocity. Due to their different inertia, in case of an emergency braking, head and torso move with a time delay between them. This is translated into a lash like effect in the neck, forcing some vertebrae in flexion at the same time that others are in hyperextension, with this being the main cervical injury mechanism. Besides the vertebrae behaviour, the role of muscles is essential in cervical injury, especially in low-speed cases.

Cervical injuries are a major public health problem with a significant social cost [1,2]. In literature, we can find numerous biomechanical studies [3,4,5,6,7] focused on the assessment of cervical behaviour. Many of these studies were performed using animals [8,9,10,11,12,13,14,15,16], crash dummies [17,18,19,20,21,22,23], full-body cadavers [12,19,20,24,25,26,27], isolated cervical [12,24,28,29,30], head–neck complexes and computational models [31,32,33,34,35,36,37].

These biomechanical models present a low biofidelity as their major weakness. We understand biofidelity as the degree of similarity with which these surrogates or models are able to represent the real behaviour of human beings. For example, in the case of dummies, they are made with more rigid materials than the organs and biological tissues. Meanwhile, post-mortem human surrogates can replicate the skeletal behaviour of humans but they lack the muscle tension. Therefore, when the focus is on the muscular response, these surrogates are not representative. There are also many studies based on sophisticated head and neck computational models [38,39,40,41,42,43,44,45,46], which successfully determine the head response [43,44,45,46,47,48,49], but they do not accurately replicate the human response as a whole, particularly in the interaction between the head and neck [49,50].

In the case in question (observation of the neck response during an emergency braking at low speed), the evaluation of the muscle behaviour with the maximum biofidelity possible is essential. The absence of biofidelity encountered in the previous biomechanical models (dummies, cadavers, or computational models) can be solved using data collected from volunteers. This is precisely one of the strengths of this work, where 18 volunteers participated. 

In addition, in the case of research with volunteers, we also have to consider that these kinds of tests are usually built inside laboratories by using sleds and restraint systems. On the one hand, to use volunteers seated in sleds does not represent the real environment of a vehicle. On the other hand, the restraint systems used (two- or four-point belts [51]) are different from commercial vehicles (3-point belts) in some cases, which limits the extrapolation of results to the real environment. To those mentioned above, it should be added that some means of transport do not have any restraint system for passengers, particularly public transport. Furthermore, autonomous vehicles designers keep in mind the possibility of travelling without restraint systems when the vehicle runs at low speed. Therefore, in the future autonomous mobility, new scenarios must be considered, such as not using safety belts.

It should be clearly understood that it is a fact that seat belts save lives at high speed. However, this restraint system may increase the risk of a neck injury at low speed, as several studies conclude [51,52]. First, it must be clarified that the reference to low speed is closely related to low deceleration since the change in velocity expected in case of emergency braking is not as great as vehicle runs at high speed. According to these studies available in the literature, the use of a restraint system during braking may amplify the injury potential due to the effect of the lap-and-shoulder seat belt resulting in the restriction of torso movement, thus leaving the free motion of the head. There is evidence, mainly via epidemiologic studies, that report an increased probability of acute neck pain and other spinal symptoms in seat-belted occupants of motor vehicles compared with non-belted passengers at low-speed impacts. 

On the other hand, some studies [6,53,54,55,56] conclude that it is not necessary to reach an impact to suffer a neck injury. Therefore, sudden braking would be enough to be a potential risk for cervical damage. Besides, keeping in mind that the number of vehicles with an autonomous braking system is increasing in the present day and driverless transport gets closer, new injury pattern studies are required, where the traffic scenario can be different since the emergency braking system would avoid the impact. This situation could lead to an increase in neck injuries, which must be carefully analysed.

Taking all that information into account, one of the aims and thus the major contribution of this work is to perform braking tests with volunteers travelling inside a real autonomous vehicle without any restraint system. In this way, the conclusions drawn by this study would contribute to understanding new possible road traffic scenarios when autonomous mobility becomes a reality, and there would be any possibility of not using safety belts. Moreover, findings could help to design a road safety system customized to the situation of not using restraint systems. 

In the following lines, the main aim is unbuilt for a better understanding of the matter. Firstly, we aim for the test performance in a real environment and not inside a lab, which, as mentioned before, may restrict the extrapolation of findings and influence the behaviour of the volunteer. Secondly, based on the position that passengers commonly adopt when they travel in real situations, it is usual to observe that when people hold a smartphone, they tilt their head forward. The issue of what would happen in this situation was then raised. For this reason, two positions were defined: seated while watching a smartphone (which means keeping the head inclined) and seated with a standard position (the latter means the back straight and leaned on the seatback). Each situation may present a different relative movement between the head and the trunk, i.e., different neck behaviour, and opens the question of if the head position adopted while watching a smartphone could involve a higher risk of suffering cervical injury than the standard seated position.

With this in mind, an experimental test, in which some low-cost devices are used, was designed. This test was focused on recording the following signals:

- Surface electromyography (sEMG) of the cervical SCM (sternocleidomastoid) and TRP (trapezius) muscles were logged using a low-cost sensor and an Arduino board before, during and after emergency braking.
- Position of the head, spine, torso, shoulders, pelvis and lower limbs were recorded by a high-speed camera and reflective markers stacked in each position.
- Acceleration measurements of the passenger and of the vehicle were registered by means of two accelerometers (a built-in smartphone and a commercial and independent system).

Eighteen volunteers were subjected to two automatic emergency braking tests inside a sensorised autonomous bus, simulating the sub-injury level. Subjects were positioned seated in the direction of travel.

A sudden deceleration situation was chosen, rather than an impact, because we intend to reproduce the future scenario of road safety. Although many people can see the autonomous vehicle as something far away, it is a fact that autonomous braking systems are already installed in commercial vehicles. Some studies [57] report different injuries compared to manual emergency braking (greater head and sternum forward excursions). On the contrary, there is also research [58] that affirms the opposite.

Bearing in mind what was previously discussed, the hypotheses considered for the present work are the following:

- First hypothesis: the usual position acquired by a passenger using a smartphone (with head inclined forwards) during an emergency braking may involve higher neck injury risk.
- Second hypothesis: no restraint system in autonomous vehicles when travelling at low speed could be safer for passengers in case of emergency braking.

It is worth emphasizing that, according to the knowledge of the authors, so far, there have been no pieces of evidence of studies evaluating cervical muscle response using smartphones while volunteers are subjected to an emergency braking in real autonomous vehicles.

## 2. Related Work

The classical hypothesis for cervical whiplash injury is the result of hyperextension movement of the neck [28,59,60,61,62,63,64,65]. When a rear impact occurs, the seat pushes forward on the trunk, while the inertia causes the head movement to be lagged. Consequently, the cervical vertebra C6 is rotated back into extension while the upper vertebrae are in flexion, causing an S shape in the cervical spine. Following, quickly, the head, to overcome the delay due to inertia, is thrown forward involving a hyperflexion neck movement. Later, other researchers investigated the role of the muscles after the impact [66,67,68], and another hypothesis was brought to the fore. They suggested that neck muscles are the site of injury, especially for low-velocity impacts. Moreover, muscle injury may occur before displacement vertebrae [6,53,54,55,56,69]. Cervical muscles contract during dynamic loads to stiffen the head–neck complex, reducing spinal motions. The time of muscle contraction varies according to occupant awareness [70,71]. Those people who know when the event will happen contract muscles prior to impact. By contrast, unaware volunteers contract neck muscles as a reflex act, which involves a time delay from the start of impact until they reach maximum contraction levels. Notwithstanding this delay, neck muscles respond quick enough to affect the head and neck motion [53,67,72]. Further, surface electromyography (sEMG) is deployed to assess the neck muscle response. The selection of neck muscles to be analysed in this study was made taking into account the following criteria: the movement that each subject was expected to perform, similar studies in the literature and the position of the muscle on the neck. In emergency braking, the mechanism of causation of neck injury involves a sudden forward motion of the head, followed by another sharp rearward movement. Sternocleidomastoid (SCM) and trapezius (TRP) muscles are generally selected as target cervical muscles to evaluate the sEMG response in low-severity impacts [43,73,74,75]. The SCM muscle bears most of the dynamic loading of the neck during rear impacts [76], in contrast to the TRP muscle, which supports most of the burden in frontal impacts [54]. 

Moreover, the equipment chosen in this work to evaluate the muscle response was surface electromyography. There are other neck muscles, such as scalenus muscle or rectus capitis lateralis muscle, however, these are deep muscles. Therefore, if we wanted to successfully evaluate these deep muscles with a low-cost device as used in this study, the best option would be to use intramuscular or needle electrodes. That is the reason why most of the previous studies consulted choose only TRP and SCM to assess neck behaviour by sEMG. 

## 3. Material and Methods

The following section describes the testbed used to carry out the experiment and collect enough information to analyse. First (Section 3.1), the study sample is defined and the different steps followed to develop the experiment are explained. Next (Section 3.2), the equipment employed is described, and finally (Section 3.3), the different analysis methods used are explained.

### 3.1. Experiment Scenarios

Eighteen healthy volunteers participated in the experiment (56% males and 44% females). All of them were considered suitable to perform the experiment because no one had any kind of injury that could result in a problem after or during the experiment. The age of the volunteers was between 22 and 54, with an average of 31.9 ± 8.8 years. Weight was between 47 and 90 kg, with an average of 66.3 ± 13.1 kg, and height was between 154 and 189 cm, with an average of 170.8 ± 9.9 cm.

**Figure 1:**
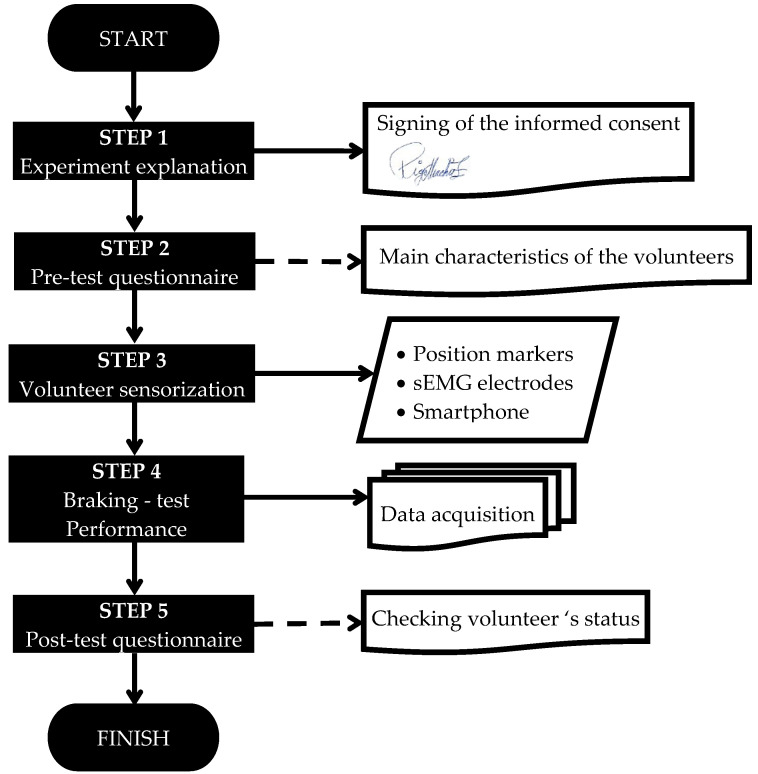
Steps of the experiment.

The following diagram (Figure 1) describes the different steps that make up the whole experiment.

Step 1 (Experiment explanation): This stage is focused on the information. In this step, every volunteer was advised about the risk of participating in the experiment, where several emergency braking scenarios would be performed. In case they agreed to take part in this study, each volunteer had to sign a consent form. In any case, the subjects always had the chance, at any time during the whole experiment, to leave the test if they wanted to.

Step 2 (Pre-test questionnaire): This pre-test survey was developed to collect the main anthropometric characteristics of the volunteers such as the height, weight, gender or age, among others. By means of this pre-test questionnaire, it was also checked if volunteers were healthy, and ensure that no one had any kind of injury that could suppose a higher lesion risk during the experiment. 

Step 3 (Volunteer sensorisation): In this stage, sEMG sensors were placed on the trapezius (TRP) and on the sternocleidomastoid (SCM) muscle, through a palpation test [77], where the mid-belly and the upper part of the muscle were located following the SENIAM (Surface ElectroMyoGraphy for the Non-Invasive Assessment of Muscles) recommendations (use of bipolar configuration with the positive electrode close to the middle of the muscle and the negative close to the distal beginning). It is important to highlight that, before sticking the sensors, the area was properly shaved with a disposable razor blade, and after, carefully cleaned with alcohol and a sterile muslin. These neck muscles and their electrode placement were chosen considering several previous biomechanical studies with a similar goal to this work [53,67,78]. Moreover, the movement of the volunteers was registered by means of reflective markers placed on the head, spine, torso, shoulder, pelvis and lower limbs, as well as a high-speed camera installed in the bus to record the passenger motion in the sagittal plane. Despite that the kinematic analysis of the movement is not part of the scope of this work, data collected by the camera was utilised to contrast the information collected by the sEMG sensors. Additionally, a smartphone was given to each of the volunteers to keep in their hands while performing one of the autonomous driving tests. Volunteers were instructed to hold the smartphone in the same manner, resting the arms on the thighs. Before starting the braking test, the research team verified that the position was adequate. In this way, the manner of holding this device was similar between different participants. Notwithstanding, authors want to point out that the real aim of using a smartphone is to assess what happens in an emergency braking when the head is initially tilted forward. 

Step 4 (Braking test performance): The deceleration test started when the volunteers were ready, they had no more questions, they had understood the experiment and when they were sensorised. The braking tests were designed following numerous references [3,53,63,67,74,75,79,80,81,82,83,84,85,86,87,88,89,90,91,92,93] and keeping in mind that subjects would not use the seat belt. The timing experiment was previously defined. Each volunteer was subjected to two different braking scenarios: Braking Test 1 (BT1) and Braking Test 2 (BT2). Both trials are explained below.

**Figure 2:**
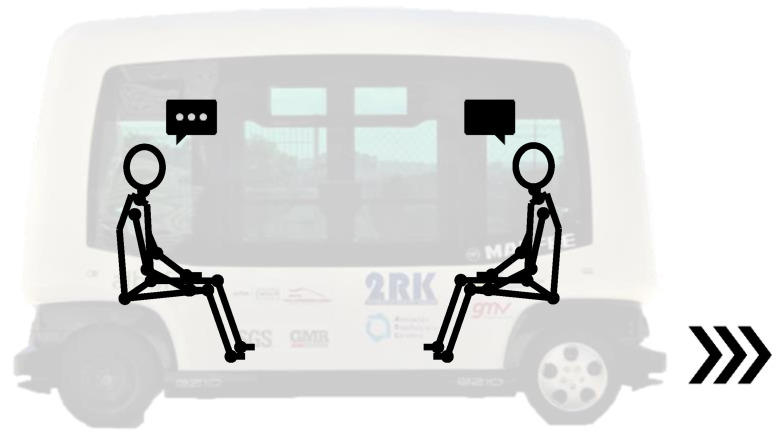
Emergency braking test 1 (BT1) schema.

**Figure 3:**
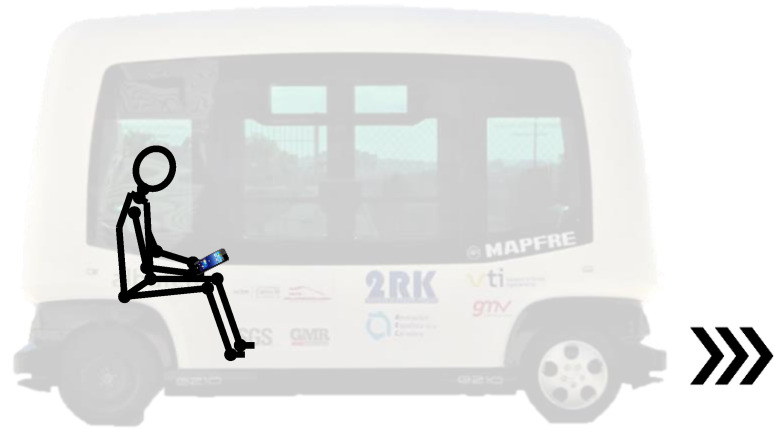
Emergency braking test 2 (BT2) schema.

- Emergency braking test 1 (BT1) (Figure 2): While the vehicle drives and the subject stays sitting in the direction of travel (meaning forward), without a seat belt and talking to one person in front of him, the autonomous bus suddenly brakes. The reasons why during this test, the volunteer is talking to a member of the research team seated in front of him, is because we wanted the muscles of the volunteer to be relaxed, since muscular tension influences the cervical response. In this way, we ensure that the participant is distracted and stress-free, as well as with the head looking ahead and not inclined. In addition, the volunteer ignored the emergency braking instant of time.
- Emergency braking test 2 (BT2) (Figure 3): While the vehicle drives and the subject stays watching the smartphone (meaning head tilted forward) sitting in the direction of travel (forward), without a seat belt and watching the smartphone between his legs, the autonomous bus suddenly brakes. In this test, it is also required that the muscles of the volunteer are relaxed. Therefore, the braking instant of time is also not known by the subject.

Timing of the experiment was programmed to reach enough velocity that allows registering a deceleration of 4 m/s2. The velocity was around 4.17 m/s (15 km/h). Finally, the time when the bus starts breaking was randomly selected in order to catch the volunteers off guard.

**Figure 4:**
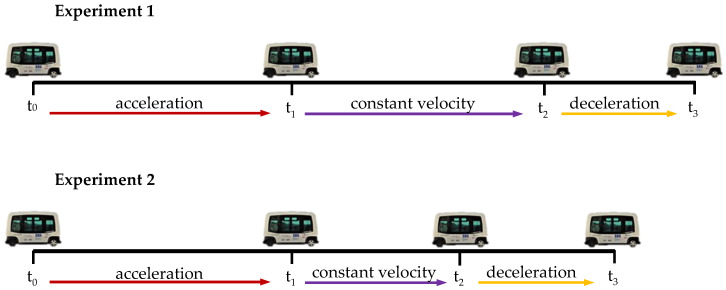
Examples of timing in two different experiments. Constant velocity (between t_1_ and t_2_) in Experiment 1 is longer than in Experiment 2. t_0_: start time (v = 0 m/s); t_1_: target velocity is reached (v = 4,17 m/s); t_2_: random braking time (v = 4,17 m/s); t_3_: finish time (v = 0 m/s).

For a better understanding of the experiment’s timing, the following figure was designed (Figure 4). As can be seen, each experiment is split into three zones, the “acceleration period” (red arrow from t0 to t1), the “constant velocity period” (purple arrow from t1 to t2) and the “deceleration time” (yellow arrow from t2 to t3). Moreover, Figure 4 shows the timing for two different tests (Experiment 1 and Experiment 2), where the first one lasts longer than the second one. That gap is due to the difference of the “constant velocity period”, because it is longer in Experiment 1 than in Experiment 2 (braking happens earlier in Experiment 2 than in Experiment 1). In contrast, the “acceleration period” and “deceleration period” were always the same, around 5 s and 2 s, respectively. The moment when the autonomous bus starts braking (t2) was randomly selected once the bus reaches enough velocity to get a 4 m/s2 deceleration to catch the volunteers off guard.

Step 5 (Post-test questionnaire): As a fifth step, we used a post-test questionnaire, where the volunteers were checked to make sure they had not suffered any kind of injury or pain during the autonomous driving test. That post-test was split into two different tests, one immediately after the braking test, and another one day after that. Checking all that information, authors could verify whether any volunteer suffered any kind of disease or injury.

Each experiment was performed only once for each subject. This decision was taken to avoid the habituation of muscles, considering some studies [59] that show habituation (that is, rapid attenuation of reflex responses) when the volunteer is subjected to several sequential stimuli. This attenuation decrements neck muscle EMG activity in 30–50% for the second or third stimulus.

### 3.2. Equipment

With the main purpose to get reliable information from the sensors during the emergency braking for the subsequent analysis of the different scenarios, several devices were used: a sEMG [94,95] system, a high-speed camera, a smartphone (with a built-in accelerometer) and a separate accelerometer. Each one focuses on the measurement of different variables. All the devices were integrated and synchronized.

**Figure 5:**
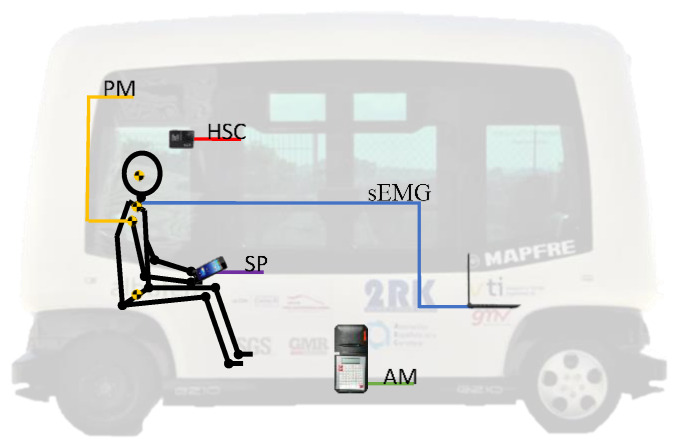
Devices built in to perform the experiment. PM: Position Markers, HSC: High-Speed Camera, SP: Smartphone, AM: Accelerometer, sEMG: sensors of surface electromyography.

Figure 5 shows the equipment used in the experiment and the position where they were installed.

- Autonomous bus: The autonomous bus used in the experiment is an EasySmile EZ10 with 6 sitting places. This bus is designed to develop smart mobility as a private or public transport, such as a driverless shuttle.
- PM: Position Markers were attached to the volunteers to follow the movement of the subject inside the autonomous bus while it brakes. This allows us to evaluate the movement of the different body parts during the experiment. The position markers were made with reflective material.
- SP: A smartphone was given to the volunteer to force him to a sitting position with the head tilted forward in BT2. Furthermore, the accelerometer built into the SP allowed us to measure the acceleration they suffered while braking in both BT1 and BT2 tests. This deceleration was measured using an application installed in the smartphone that registered the triaxial acceleration signal.
- sEMG: The EMG system employed in the present experiment was used and validated in the past by the authors of this work [94,95]. This device is made up of an Arduino Mega board and a sEMG low-cost sensor. The low-cost sensor is connected, employing three wires. At the end of each wire, there is an adhesive electrode that must be placed on different points of the muscles (on the middle of the muscle, on the beginning and close to a bony area as a reference point). The whole device was plugged into a personal computer and implemented by means of Simulink and Matlab [96]. Technical information related to the whole sEMG device can be found in Table 1.
- HSC: A High-Speed Camera was installed on the window of the autonomous bus located to the right side of the volunteer. To get a reliable video recording, it is important to avoid the vibration of the camera during braking. Therefore, it is essential to fix it properly to the bus, as well as selecting a camera model with image stabilisation. The camera must also guarantee a low image distortion and a capture of a high rate of frames per second. Moreover, it must be a portable system and easy to install. The model selected for the experiment has all those characteristics and it can also be remotely controlled. Moreover, it is powered by rechargeable batteries. The main technical information is summarised in Table 2.
- AM: The accelerometer used to measure the deceleration of the vehicle during the braking tests was installed close to the centre of gravity of the autonomous bus and attached to the floor of the vehicle. The accelerometer was calibrated before the test, and the accuracy and more technical information of it can be found in the following table (Table 3).

### 3.3. Analysis Method

Data from all the devices were registered during the whole experiment and it is important to process it in order to analyse it. First, the synchronisation process was performed. After that, the sEMG signal was trimmed to be filtrated and, later, normalised.

The synchronisation was developed employing different triggers that create a peak in each sensor’s signal. The first trigger synchronised the signal between the sEMG sensors and the smartphone, and the second one synchronised the signal between the smartphone and the accelerometer fixed on the floor of the vehicle. 

Every volunteer had to perform two different movements to obtain those peaks. Firstly, each volunteer stood up roughly, and secondly, they had to stamp their foot on the ground. As a result, the first peak in the smartphone and in the sEMG signals was registered, and a second peak in the smartphone and accelerometer placed on the floor.

**Figure 6:**
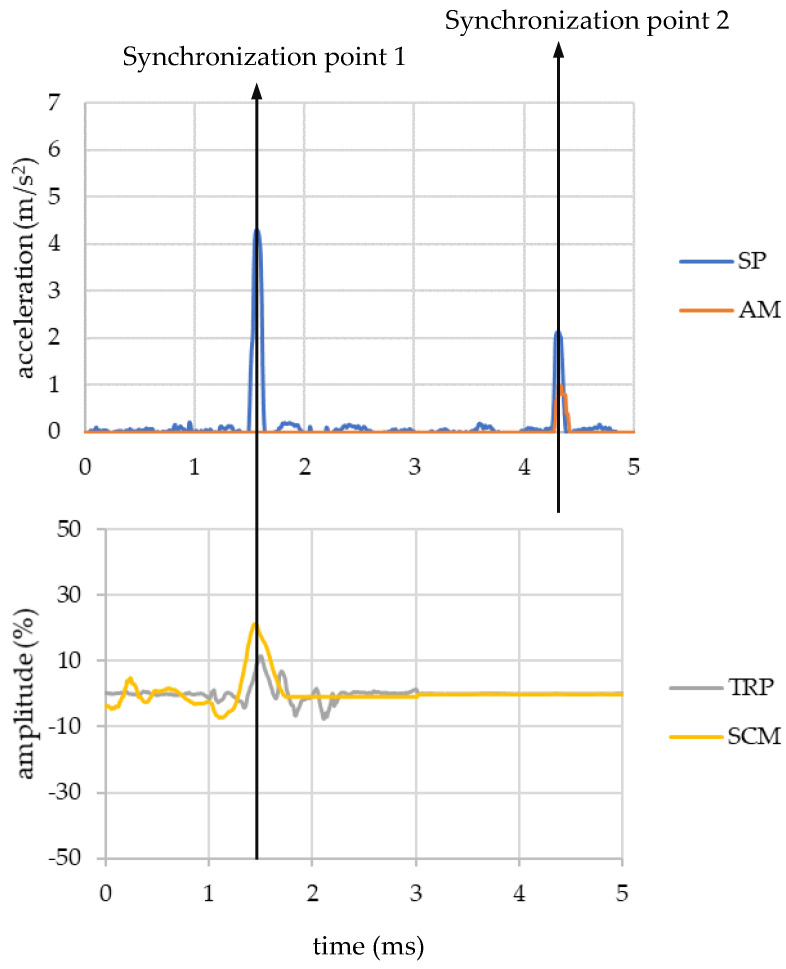
Synchronisation timing of the signals employing the two synchronisation points.

The process has been summarised in the following figure (Figure 6), where the beginning of the four signals (SP, AM, TRP and SCM) are plotted. On the top, the signals of the SP and AM are represented and the sEMG signal from both muscles is shown on the bottom. As it was said above, the first movement originates a peak in the SP and in the muscles (Synchronization point 1) that makes it possible to synchronise both signals. After that, the second movement is performed, and it produces a common peak in the SP signal and in the AM signal. In this way, all signals were synchronised. It is important to mention that this entire process was carried out with the bus stopped.

According to the segmentation of the signal, the process was developed after the synchronisation, and considering the main analysis of the experiment, the muscle behaviour when the bus suddenly brakes. Therefore, the section that was trimmed is the one belonging to the muscular activity, defined by means of the changes in the accelerations measured.

The analysis method used for every signal was different. For this reason, the following section is split into different subsections.

#### 3.3.1. Acceleration Data

Acceleration data was captured by two different devices (AM and SP). It should be clarified that AM had a main aim of verifying that the deceleration during the tests complied with the value initially programmed for the bus braking. In this way, we assured that no volunteer was subjected to a deceleration level which could endanger human health. On the other hand, the acceleration signal from the SP was used to know the maximum value of deceleration, in the location where the smartphone was held by the volunteers, to verify that the experiment did not overtake high values of acceleration which could imply a potential risk of injury. Both signals were compared to define possible differences between the acceleration of the bus and the acceleration that the passenger suffers during the emergency braking. The synchronisation process between both signals has already been explained above. 

#### 3.3.2. sEMG Signal

The sEMG signal was captured from TRP and SCM muscles. Unfortunately, the sEMG signal is usually affected by noise, which can cause a wrong understanding of the signal. Therefore, to guarantee a good interpretation of the behaviour, all data acquired by the low-cost system was filtered [97,98,99].

After that, the sEMG signals were normalised to be able to carry out the comparison between all of them [100]. Different ways can be undergone, using the Maximal Voluntary Contraction (MVC) [101], one of the most widespread methods, but this is inappropriate when dynamic movements are studied [102], or utilising the maximum value of the task [100], among others. Unfortunately, none of these methods fit this experiment. The first one did not fit because a bad performance of the MVC exercise could cause some kind of stress in the muscles [103] that could turn into an injury after the experiment (several emergency braking tests without a seat belt). The second one, for its part, because this method has the disadvantage of being based on individuals and not in the maximum capacity of the muscle [100]. For that reason, the normalisation developed in the present study was done employing the mean activation levels obtained during the task [104]. This method makes possible the comparison of muscle signal patterns between different subjects [105,106] and without risk of injury.

The way to proceed to normalise the signal was first the segmentation of the signal corresponding to the braking time; next, the filtration, and, finally, the assessment of the mean value to normalise it. To carry it out, the signal was only considered while the autonomous bus was braking. In other words, the signal between the time when the brake starts and ends. Finally, the normalisation was carried out using this parameter. It is important to highlight that each signal was normalised individually using the mean parameter assessed on each volunteer’s experiment.

The analysis used in the experiment, and developed by different scripts with Matlab [96], was split into the following stages:

- Signal filtration: This step aimed to remove the noise of the signal. It was done in two different steps. Firstly, the background noise was identified, and then, the main noise of the signal was identified based on an evaluation of the Fast Fourier Transform [94,107]. Secondly, the signal was filtered by a bandpass Butterworth (40–100 Hz, order 4) filter, which was used to remove the main noise from the signal and a stopband Butterworth (45–55 Hz, order 4) filter because some noise centred around 50 Hz was addressed.
- Parameter assessment: When the signal was cleaned, the last step was the assessment of the signal behaviour parameters. To that aim, a script in Matlab was written and run individually. The script was coded to calculate the next variables:
  - Amplitude of the TRP’s and SCM’s signals during the emergency braking.
  - Instant of time of the muscle activation, to see which one starts working first when the bus suddenly brakes.
  - Maximum sEMG peak of the TRP and SCM during the emergency braking.

#### 3.3.3. Position Markers

Markers were used to follow several points of the subject, from the head to the hip, during the tests. By using video photogrammetry software, the movement of the volunteer inside the autonomous bus during the braking time was assessed, in this way obtaining the position of markers for each frame.

The position of the makers was later assessed through several scripts in Matlab [96], making it possible to register the movement evolution along the time. The trajectory of all these markers was obtained to know the range of movement for each volunteer and each test. Moreover, special attention was paid to the relative movement observed between head and torso during braking, since it has long been assumed that head displacement to the trunk is an element of the cervical injury risk. Consequently, if a lesser relative movement between these body areas is observed, it suggests that the conditions used in that test may be protective.

Later findings drawn from videos allowed us to conclude what situation could be less harmful to the neck in case of emergency braking. This information was also contrasted with the sEMG response. In the latter case (sEMG signal), a greater muscular response may involve a riskier situation in the cervical spine.

## 4. Results

Turning now to the experimental results, the next section is divided into three parts. Each part presents the results of every device. The first one from the accelerometers, the second one from the sEMG sensors and the third one from the movement of the volunteers during braking.

### 4.1. Accelerations

The autonomous bus was programmed to reach a deceleration of 4 m/s^2^. This acceleration value was chosen according to diverse studies [51,54,56,59,75,76,79,91,92,108] where low speed pre-impact tests were performed using volunteers. In these studies, seated and stabilised subjects were exposed to sled accelerations ranging from 4 to 12 m/s^2^. Nevertheless, in all these cases available in the literature, volunteers used restraint systems. Therefore, they can support greater values of acceleration. However, in our work, all volunteers performed tests without retention. That is the reason why we only decided to include the minimum value used in the mentioned works (4 m/s^2^) to prevent possible damage during tests. It should be noted that the threshold of acceleration that can be harmful depends on the subject. Gender, age, physical condition, previous neck injuries, among other factors, may affect the behaviour of the volunteer during braking. For example, females have lower muscle strength. However, females show greater neck flexibility. Consequently, some subjects could support higher acceleration without expecting any neck pain. Despite this, we decided to assume the least risk for our participants, so we chose the minimum value of deceleration.

**Figure 7:**
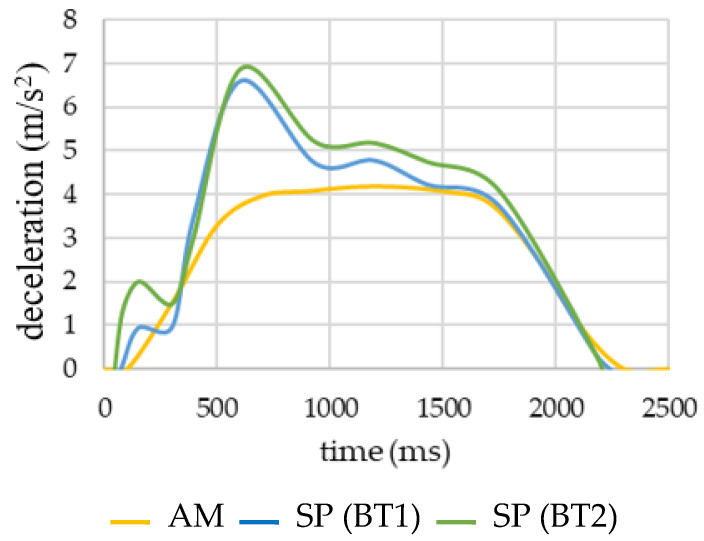
Average acceleration registered by the AM (accelerometer) and SP (smartphone) in the experiment. The deceleration results are absolute values.

Figure 7 represents the average of the acceleration registered in all the experiments. In Figure 7, it can be noticed that the SP signal was split into two different ones. The first one corresponds to the mean of the values obtained in BT1 and the second one to the mean of the results obtained in BT2. In this graph, it can also be appreciated that the acceleration that each volunteer experienced inside the autonomous bus is greater than the acceleration that the vehicle underwent when the emergency braking was activated.

### 4.2. sEMG Signals

sEMG signal was acquired in parallel from two different muscles, an agonist and an antagonist, depending on the movement of the volunteer.

All the signals have been trimmed to visualise critical timing (from the rest to the maximum displacement of the volunteer) when the emergency braking happens.

**Figure 8:**
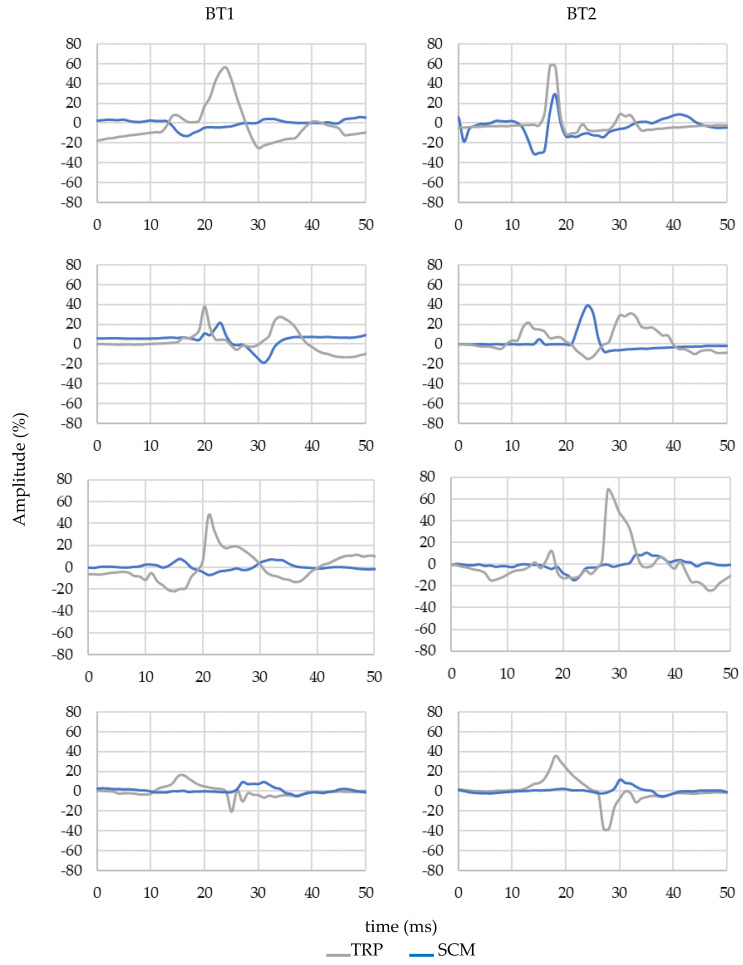
Normalised sEMG signal of four volunteers from the SCM and TRP muscle. First row: Man, 31 years old, 185 cm height, 70 kg. Second row: Woman, 41 years old, 172 cm height, 78 kg. Third row: Man, 39 years old, 189 cm height, 80 kg. Fourth row: Woman, 32 years old, 162 cm height, 47 kg. Left column: Emergency test braking 1. Right column: Emergency test braking 2.

Figure 8 shows an example of the signals obtained from four different volunteers. The left column corresponds to the braking test 1 (BT1) and the right column corresponds to the braking test 2 (BT2). Moreover, the signal from both muscles is represented: the TRP response is represented in grey and in blue is the SCM response.

The summary of the information collected from all the volunteers has been summed up in the following table (Table 4). Results have been split into the genre (♀ (women) and ♂ (men)) and it includes the average value (µ) and standard deviation (σ). Both values were assessed for men and women separately and considering the amplitude of the signal and the maximum value once it had been normalised. The P.P.O.M (Percentage of People Over the Mean) parameter was also included. It represents the percentage of people (♀ or ♂) who registered a mean value during the braking time that exceeds the µ value for its corresponding genre. 

Apart from that, data from the sEMG device was also sorted by age and gender (Table 5) gathered in both experiments (BT1 and BT2). In the same table, the average value (µ) and standard deviation (σ) from the amplitude of the signal and maximum value can be consulted.

For the dependent variables (TRP amplitude, SCM amplitude, TRP max and SCM max), a multivariate analysis of variance (MANOVA) was used to assess differences related to gender and age. All tests were performed using a significance level of α = 0.05.

According to the MANOVA statistics, in BT1, age has a statistically significant relation with the sEMG response. Age is the most significant factor (*p*-value *<* 0.0001; Wilks’ lambda = 0.239431) for the SCM amplitude and TRP amplitude variable. For the SCM maximum and TRP maximum values, age is also the most significant effect (*p*-value < 0.001; Wilks’ lambda = 0.308732). On the other hand, in the case of BT2, the same behaviour is also observed. The most statistically significant factor is the age for SCM amplitude and TRP amplitude variables (*p*-value < 0.0001; Wilks’ lambda = 0.177472). For the SCM maximum and TRP maximum values, age remains to be the most significant factor also in BT2 (*p*-value *<* 0.0001; Wilks’ lambda = 0.255436).

### 4.3. Position Markers

Position Markers allow following the head and torso movement employing the high-speed camera. It makes it possible to analyse the situation and see the neck motion range. 

**Figure 9:**
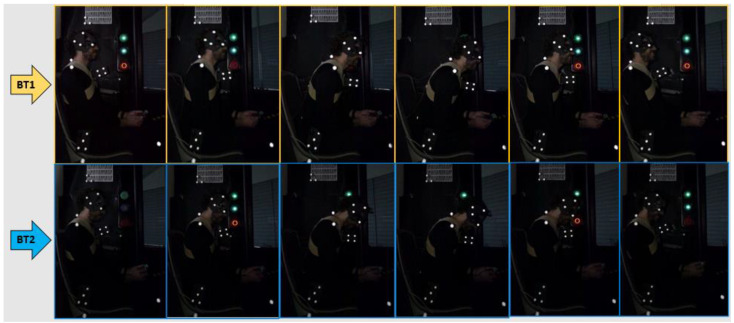
Movement of one volunteer during BT1 and BT2.

Figure 9 shows some frames of the movement of one volunteer during both tests (BT1 and BT2) recorded by the HSC. All videos were processed by a software of video analysis of movement to track the position of markers during braking. Then, these positions values were analysed by a script in Matlab. 

**Figure 10:**
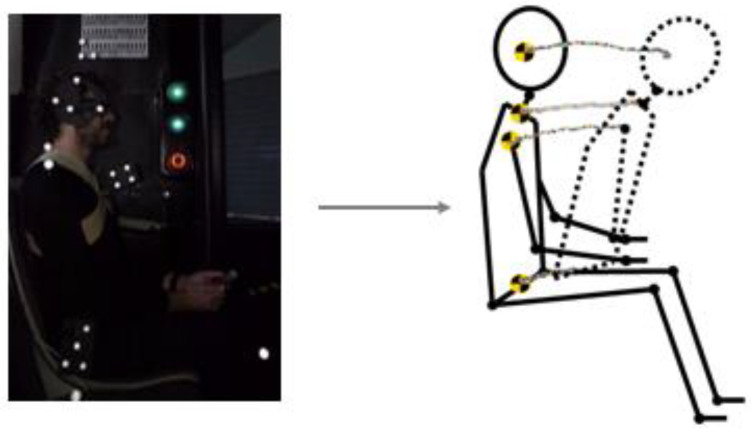
The trajectory of position markers of a volunteer during BT1.

In one example of the data, after analysing by Matlab, as can be seen in the following figure (Figure 10), where through the trajectory of the position markers, the whole movement range of one volunteer is plotted.

**Figure 11:**
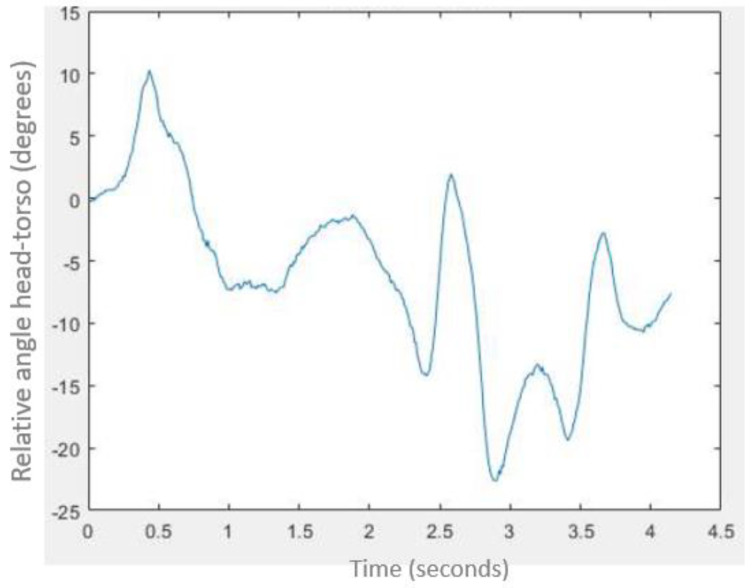
Relative angle between head and torso during BT1.

Relative movement between head and torso was also obtained utilising another script in Matlab. In Figure 11, we can see an example of this.

Based on the trajectory of Position Markers and the analysis of the relative movement, an average relative angle of 25° approximately between head and torso was observed when the volunteer is travelling looking forward (BT1). However, in the case of BT2 (when the passenger is watching a smartphone), the volunteer starts the test from a position in which there is already an initial relative angle between head and torso up to a value of 45° in many cases. In this second test, it was observed that most of the volunteers moved as a block, that is, without experiencing a significant relative angle of the head concerning the torso, with this angle always being lower than observed in BT1.

## 5. Discussions and Conclusions

The cervical spine is one of the most sensitive body regions to sudden changes in velocity. Therefore, when the collision is not consummated, involving only abrupt deceleration, as occurs during an emergency braking, it is fundamental to evaluate the muscle response by means of biomechanical models capable of preserving the biofidelity of the muscle. This requirement of muscular biofidelity is met by using volunteers, as is the case of this study, where 18 participants were subjected to two different automatic emergency braking tests while they were sitting inside a real autonomous vehicle (in this case, a bus). The procedure followed in this work can serve as an example to perform reliable biomechanical tests, under real conditions, with low-cost devices.

Two situations were included in the test scenarios. On the one hand, we incorporated the normal use of the smartphone during the travel, due to several reasons: its frequent use in journeys by public transport, its use generally modifies the head position, and consequently, influences the movement in case of braking. On the other hand, we characterised our driving test without seat belts, because there are studies that conclude that the restraint system may involve higher neck injury risk. It should be highlighted that all trials were performed inside a real autonomous vehicle, for the purpose of keeping volunteers in a real environment. 

Considering that the future of the autonomous mobility is increasingly close for citizens, there is a need to assess new possible road traffic scenarios, where not only may passengers not use safety belts, but also do not need to drive the vehicle and, therefore, they can do other activities such as consulting the smartphone or other devices. This is precisely the main strength of this work, to evaluate what could happen in these kinds of situations, all evaluated in a real environment. Bearing in mind the aforementioned factors, an experiment was designed with the purpose of evaluating the potential risks that a passenger would face in the event of emergency braking inside an autonomous vehicle.

This experiment was divided into several steps, among them: explanation to volunteers about the experiment and its risks, sensorisation of the volunteers, emergency braking performance for both cases and later, analysis of the sample. In order to carry out the experiment, it was necessary to use several devices: a low-cost sEMG system, different accelerometers and a photometric measurement system made up of a camera and several markers.

Turning now to the experimental evidence on the tests, the first thing that is going to be dealt with are the accelerations. The signal from AM allowed us to verify that the deceleration value initially programmed for the vehicle braking (4 m/s^2^) was not exceeded. In this way, we ensured that volunteers were not subjected to risky deceleration values. Comparing acceleration signals, it could be said that there is an important gap between the signal acquired by the smartphone (SP) and the accelerometer (AM) (Figure 7). This gap, characterised by the maximum peaks, is approximately 1.5 times greater in the location where the smartphone is than in the autonomous bus. Therefore, although the deceleration of the autonomous bus is not mightily high, the deceleration suffered by the passengers may be significantly greater. As a conclusion, when maximum levels of deceleration are programmed in future autonomous vehicles, this fact must be considered. This may be explained by the inertia of passengers travelling without safety belts, experiencing free movement. It should be clarified that the acceleration registered by the SP corresponds to the location where the smartphone is held, that is, in the hands of the volunteers. It must be emphasised that this acceleration registered by the SP does not necessarily correspond with the acceleration value of the head. As we have already mentioned, participants were instructed to position the SP in the same manner, as well as the research team verified in the videos that in any case, the volunteer brought the hands forward. By contrast, it can be observed in the videos that the head shows a greater range of motion during braking. As a consequence, the acceleration suffered by the head may be even greater than the value registered in the SP. In addition, the relative movement of the brain inside the skull should not be forgotten, nor should the high sensitivity of cervical muscles to sudden changes of velocity. Therefore, when the emergency braking for an automatic system is designed, it must be considered that the value of acceleration initially defined for the vehicle may imply a greater risk of cervical injury than expected for the corresponding initial value of deceleration exercised by the bus, because it is proven that acceleration peaks suffered by passengers are higher than those established for the bus braking.

Moving on now to consider the sEMG signal, the behaviour between muscles (Figure 8) is completely different. In emergency braking, the mechanism of causation of neck injury involves a sudden forward motion of the head, followed by another sharp rearward movement. Due to its inertia, the head is delayed concerning the torso movement. This is the basis of the injury mechanism in the neck in these cases. A forced forward movement of the head activates the trapezius first to control or resist this motion. In contrast, the sternocleidomastoid tends to be more involved in controlling or resisting the head motion backwards during the rebound phase.

On the basis of sEMG signals obtained, it can be observed that the TRP muscle acts before the SCM muscle in the forward travel direction when braking occurs. This is a common behaviour in this type of braking [43,66,67,68,75], where the passenger travels forward. In addition, if Table 4 is analysed, it can be seen how the amplitude values obtained in the case of BT2, for men and women, are lower than in BT1. Therefore, we can assume a lower-risk situation regarding cervical injury in case of BT2. Apart from that, what Table 4 confirms is that the behaviour of the TRP, compared with the SCM muscle, is completely different, observing this for both genres. TRP muscle does not only have a higher amplitude (in BT1 and BT2) but also, the maximum values are always higher than the SCM muscle. This response (TRP muscle is more active) is usually registered in this kind of braking test [43,66,67,68,75], where passengers are positioned in the direction of travel during forward impacts. On the other hand, if we consider only the gender, there are no statistically significant differences (*p*-value > 0.05) between mean values for females and males. By contrast, when the age factor is included in the analysis, the differences between genres may be then appreciated. In that case, it is observed that males, younger than or equal to 35 years old, show higher sEMG response for SCM and TRP muscles. This can be seen both in the results of the BT1 and BT2 experiments. On the contrary, when subjects are older than 35, females show slightly higher values for both muscles in both experiments (as we can see in Table 5). Notwithstanding the above, a statistically significant difference according to the genre was not observed.

Additionally, and mainly due to the flexibility of the women, a greater percentage of women who moderately overtake the mean of the amplitude and the mean of the maximum sEMG value was observed. Thus if the P.P.O.M. parameter (Table 4) related to the amplitude of the TRP muscle in the BT1 test is observed, there is a 2.5% difference between men and women (60% of men and 62.5% of women), and related to the SCM, the difference increases to 10% (40% of men and 50% of women). In contrast, for BT2, there is a higher difference in the TRP muscle (50% of men and 62.5% of women) and quite a bit higher in men than women for the SCM muscle (70% of men and 50% of women).

On the other hand, when subjects are younger than or equal to 35 years old, males show higher maximum values than females for both muscles in the two experiments. However, when subjects are older than 35, in the case of BT1, females show greater maximum values than males for TRP muscle, and males show higher maximum values than females for SCM muscle. For the BT2 experiment, and when subjects are older than 35, females and males deliver similar maximum values for both muscles. Consequently, it is not possible to establish a statistically significant difference considering only the genre.

If the maximum values are analysed according to the P.P.O.M. parameter, the same behaviour can be seen: the percentage of women who overtake the mean value is higher in all the cases, except in the BT2 experiment and SCM muscles, where the percentage of women stands under the percentage of men (50% of men and 37.5% of women). Moreover, if Table 5 is observed, where volunteers were sorted by age and gender, it can be seen that independently of the genre, as the age of the volunteers increases, the amplitude of the signal from the TRP and SCM also rises. People over the age of 35 double this value. The same behaviour is also observed in the maximum values (that is, people aged under 35 have smaller peaks than people over 35). In fact, results from MANOVA analysis show that age is the most statistically significant factor that affects the sEMG response (*p*-value < 0.0001) both for TRP and SCM muscles. In conclusion, the age of the passenger is an important factor to be considered when road safety systems are designed.

All of that is confirmed by the motion analysis, carried out thanks to the Position Makers (PM) and recordings by the high-speed camera. In this way, the relative movement between the head and torso can be observed. Comparing the videos of BT1 (volunteers started with a neutral forward-seated position while they talked to the passenger seated in front of them, face to face) to the videos of the BT2 (volunteers started with a voluntary forward-seated position while they used a smartphone), it is observed that in the second test (BT2), all volunteers start with the head bent regarding the torso. This starting relative angle in BT2 reaches, indeed, a value of 45° in many cases. This initial relative angle between head and torso may modify the relative movement between these body parts during braking tests, and therefore the sEMG response and the risk of a neck injury. In all these second tests, it has been noted that most of the volunteers moved as a block during the emergency braking. That means subjects did not experience a significant relative angle of the head with respect to the torso in BT2. In any case, this relative angle observed during BT2 was always lower than that observed in BT1. If we assume that a sharp movement of the head for the torso and its consequent relative angle involves more risk in case of cervical injury, the BT2 scenario may be protective in case of emergency braking according to our results. 

In view of the above, and to assess our first hypothesis (if using the smartphone during an emergency braking may involve higher neck injury risk), results of all volunteers from both emergency braking tests (BT1 and BT2) were compared. In conclusion, when volunteers held the smartphone (BT2), they start the test with a bent position between head and torso which modifies their neck behaviour during the emergency braking. Based on our results, if the head is flexed with respect to the torso at the time of the emergency braking, cervical injury risk diminishes, by exhibiting a lesser relative angle during braking. Moreover, this has been reflected in the sEMG response. Using the smartphone, both TRP and SCM cervical muscles registered a lower magnitude of the sEMG signal. These findings fit with conclusions obtained by other researchers [75], where a lower head displacement is observed where the head and trunk are flexed during sled deceleration tests. 

Finally, with the aim to check our second hypothesis (no restraint system in autonomous vehicles when travelling at low speed could be safer for passengers), our findings were analysed also taking into account conclusions obtained in the literature, where tests using seat belts with the same deceleration levels (approximately 4m/s^2^ [51,54,56,59,75,76,79,91,92,108]) were performed. The findings drawn from the sEMG signals and the relative motion observed between head and torso during braking were contrasted. Checking our recordings, it was observed that all volunteers showed a larger whole-body excursion during braking compared to results found in the literature. In these other studies, the torso of volunteers does not separate from the seat during deceleration due to the restraint system, while the head moves suddenly forward. In our case, the torso, since it was not belted, accompanied the movement of the head during braking. As a result, in our tests without retention, we also found lower relative movement between head and torso during the body excursion compared to research with seat belts. It has long been assumed that the more relative motion of the head with respect to the torso, the more possibility to lead to a higher cervical injury risk. Therefore, it can be concluded that, based on our results, a restraint system may involve a riskier environment regarding cervical injury when travelling at low speed, and the explanation may lie in the movement of the torso. When a restraint system is used, the trunk is stopped at the braking time while the head continues moving forward, causing greater relative movement than without a seat belt. The latter means higher loads in cervical muscles, and therefore a higher sEMG response. Notwithstanding, it should be pointed out that these conclusions cannot be extended to high-speed values. Even then, this study, by performing braking tests with volunteers travelling inside a real autonomous vehicle without any restraint system and using a smartphone, may contribute to understanding possible road traffic scenarios and the associated cervical injury patterns during an emergency braking when the autonomous mobility becomes a reality.

It is important to highlight that the development of the experiment was always focused on the health of the subjects and always keeping in mind ways to keep them safe. Other limitations of this study should also be noted. For example, in this work, only two main neck muscles were analysed. It may be therefore advisable to assess deeper neck muscles in a future study; but for this, other methodology different to surface EMG should be used to get a good signal from these muscles.

It should be emphasised that the aim of this study was focused on mainly analysing the influence of the body position during the braking. There are studies [109,110] which conclude that seating position while travelling affects the risk of cervical injury in case of an accident more than age and genre. Therefore, it could be interesting to repeat these experiments with a broader sample of volunteers as future work to obtain a deeper analysis targeted to factors like age or genre.

## Figures and Tables

**Table 1:** Technical information of the sEMG device.

Arduino Mega		sEMG Low-Cost Sensor	
Microcontroller	ATmega2560	Shape/size (exclude grip)	Round/ ⦰ 24 mm
Vin (V)	7–12	Gel/adhesive/sensor area	201/251/80 mm^2^
Vout (V)	6–20	Gel characteristics	Conductive hydrogel
Analogue Inputs	16	Sensor	Polymer Ag/AgCl
Sampling rate (Hz)	1000	Bandwidth (Hz)	10–400
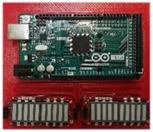			

**Table 2:** Technical information about the High-Speed Camera used.

**Camera Model**	**SONY DSC-RX0**	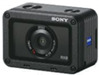
Sensor type	Sensor CMOS Exmor RS type 1.0 (13.2 mm × 8.8 mm), 3:2	
Megapixels	21.0	
Dimensions	59 mm × 29.8 mm × 40.5 mm	
Lens type	Lens ZEISS Tessar T*	
Ultra-slow motion	Up to 960/1000 fps	

**Table 3:** Technical information of the accelerometer MAHA VZM 300.

**Accelerometer model**	**MAHA VZM 300**	** 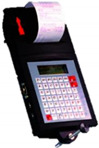 **
Measuring range	0 m/s²–20 m/s²	
Internal power supply	6 V/1.8 Ah	
Measurement accuracy	±1%	
Data rate (Hz)	100	
Dimensions	245 mm × 124 mm × 55 mm	

**Table 4:** Summary of the sEMG data from the experiments: Braking Test 1 (BT1) and Braking Test 2 (BT2). P.P.O.M: Percentage of People Over the Mean, (µ) average value and (σ) standard deviation.

Genre	Signal	Muscle	BT1			BT2		
			µ	σ	P.P.O.M	µ	σ	P.P.O.M
♂	**Amplitude**	**TRP**	101.9	49.3	60.0	75.3	60.0	50.0
		**SCM**	62.6	55.8	40.0	44.6	34.8	70.0
♀	**Amplitude**	**TRP**	107.1	59.7	62.5	75.9	56.2	62.5
		**SCM**	63.2	46.9	50.0	55.1	29.1	50.0
♂	**Max**	**TRP**	51.1	33.3	30.0	50.3	44.6	50.0
		**SCM**	28.3	29.4	40.0	27.7	31.3	50.0
♀	**Ma** **x**	**TRP**	44.4	34.1	37.5	49.1	42.0	50.0
		**SCM**	25.7	16.3	50.0	33.6	27.7	37.5

**Table 5:** Summary of the sEMG data from the experiments sorted by age and gender: Braking Test 1 (BT1) and Braking Test 2 (BT2).

			♀							
			**BT1**				**BT2**			
		**Age**	**≤35**		**>35**		**≤35**		**>35**	
			**µ**	**σ**	**µ**	**σ**	**µ**	**σ**	**µ**	**σ**
%	Amplitude	TRP	81.92	46.01	123.78	67.29	52.08	42.75	113.99	26.78
		SCM	62.07	38.53	133.30	49.72	27.85	20.29	72.63	27.68
	Max	TRP	21.73	12.50	150.87	29.03	29.00	26.57	85.74	19.91
		SCM	28.75	16.31	87.65	15.39	11.84	8.34	54.15	38.72
			♂							
			**BT1**				**BT2**			
		**Age**	**≤35**		**>35**		**≤35**		**>35**	
			**µ**	**σ**	**µ**	**σ**	**µ**	**σ**	**µ**	**σ**
%	Amplitude	TRP	102.93	65.16	97.89	23.13	66.41	53.00	116.54	6.63
		SCM	76.13	53.97	109.23	11.09	63.98	34.72	61.45	51.18
	Max	TRP	48.98	35.74	119.52	16.41	36.69	31.44	94.29	36.97
		SCM	34.28	27.11	130.34	6.31	34.49	20.17	53.81	61.35
